# Losses of expression of the antigens A, Le^a ^and Le^x ^and over-expression of Le^y ^in carcinomas and HG-SIL of the uterine cervix

**DOI:** 10.1186/1746-1596-3-38

**Published:** 2008-09-11

**Authors:** Ernesto Moro-Rodríguez, Emilio Álvarez-Fernández

**Affiliations:** 1 Área de Patología. Universidad Rey Juan Carlos, Madrid, Spain; 2 Servicio de Anatomía Patológica. Hospital General Universitario Gregorio Marañón, Madrid, Spain

## Abstract

**Background:**

The glycosylation of a great number of molecules, glyco-protein or glycolipids, has been of interest for decades.

**Objective:**

To compare the expressive patterns of the isoantigenic determinants of histo-blood groups ABH and Lewis in squamous and simple epithelium and in precursors and cancers of the cervix.

**Methods:**

A total of 36 lesions and neoplasms (10 LG-SIL, 16 HG-SIL and 10 invasive carcinomas) have been studied with immunohistochemical techniques, using monoclonal antibodies (MoAb BG1 to BG8) for precursor chains, blood-group ABH and Lewis group Le^a^, Le^b^, Le^x^, and Le^y^, and four types of lectins. In addition, we have studied the expression of p53 protein and PCNA, establishing the rate of proliferation of each lesion. Using PCR techniques, we have also detected part of the intron of the E6 gene of HPV-16.

**Results:**

In the invasive cervical carcinomas, we observed a loss of expression of the Le^x ^antigen (p < 0.01). With regard to the progression of the different lesions studied, we found alterations in the patterns of expression of the antigens of the ABH and Lewis blood groups. There was a tendency towards a loss of expression and heterogeneous patterns in the more advanced lesions, as well as over-expression of the Le^y ^antigens. With PCNA, we established a proliferative rate which tended to be greater in relation to the progression of the cervix neoplasms.

**Conclusion:**

These results indicate that there is a relation between the losses of histo-blood groups and the progression of the squamous intraepithelial lesions.

## Background

The glycosylation of a great number of molecules, glyco-protein or glycolipids, has been of interest for decades. Increasing evidence highlights the fact that the carbohydrates located in the cellular surface and in the extracelular matrix play an important role in cellular recognition and in the organization of tissues and organs, and that the aberrant expression of carbohydrates is closely related to certain diseases and to the progression of neoplasms. Historically, the functional role of carbohydrates in cellular recognition has been the most clearly demonstrable evidence in the development process and in cellular differentiation [1].

Most of the patterns of peripheral/terminal glycosylation is polymorphic in humans and constitute what is known as histo-blood groups [2], among which we know the systems ABO, secretor, Lewis, TTn and P. These antigen determinants have been identified in most human epithelia [3,4].

Furthermore, in some neoplasms there are frequent alterations of the cellular surface glycoconjugates that, in general, have been classified in: (A) incomplete synthesis, with or without accumulation of precursors, (B) synthesis of neoglycolipids and (C) organizational changes of the glycolipids in cellular membranes [5].

They are many studies that relate the alteration of the glycoconjugates in different human neoplasms, and the works dealing with the human urinary tract [6-8], the digestive tract [9-11], the lungs [12-16], or the genital organs [17-22] are currently classics.

In addition, several molecular biology techniques arise as powerful tools that could provide complementary and valuable information to immunohistochemistry and to the traditional morphologic diagnosis. In this work we have combined a immunohistochemical study for the expression of PCNA (a known proliferative marker-PC-10 clone) and p53 (DO7 clone) with results of amplification of part of the E6 intron of the HPV-16.

PCNA is a nuclear protein of 36-kd cofactor of the polimerase delta detected in interfase cells (G1, S and G2) [23,24]. Mutations of p53 are the most frecuently reported in human neoplasms [25]. It has been suggested that p53 intervenes in the control of the cellular growth in the critical point among the phase G1 and S playing an important role of "guardian of the genoma". p53 could prevent the progression of cells through the cell cycle when DNA damage has occurred. Thus allows DNA to repair [26]. With regard to the influence of p53 upon several promoters it is considered that p53 could downregulate the PCNA RNAm [27]. Its contingent lost of function would have as consequence an increase of the cellular proliferation. Some authors also have observed that p53 would regulate the transcription of p21 protein which is known that can inhibit directly the PCNA and other cyclins impeding the synthesis of DNA in case of cellular damage [28,29]. Finally it is known that a HPV protein coded in the E6 gene could block and promote the degradation of the wild type of p53 [30,31]. Consequently the degradation of p53 could decrease p21 releasing its inhibition on PCNA.

We consider that the cervical intraepithelial neoplasms and carcinomas of the uterine cervix represent an optimum model for the study of the phenotypic alterations in the progression towards malignancy although, up until this moment they have been insufficiently explored. For this, reason, we have studied a total of 36 lesions that covered a spectrum from cervical metaplasias with and without dysplasia and/or condyloma to the invasive carcinoma, focusing our attention on the possible alterations of their patterns of glycosylation.

## Methods

### Patients and lesions included

Samples of cervical lesions and normal adjacent tissues were surgically obtained from 30 patients (22 total hysterectomies, 8 cone resections and 3 biopsies). Among these cases we found the following lesions: 19 mature metaplasia, 1 immature metaplasia, 1 regenerative epithelium, 9 low grade squamous intraepithelial lesions (6 flat condylomas, 2 condylomas acuminata, and 1 CIN-I), 16 high grade squamous intraepithelial lesions (3 CIN-II and 13 CIN-III), and 10 invasive carcinomas (9 squamous carcinomas and a mixed-carcinoma-epidermoid and adenocarcinoma-).

### Immunohistochemistry

Five μm thick sections of formalin-fixed and paraffin-embedded tumor specimens were cut and deparaffinized according to routine histological techniques. Serial sections were stained with primary monoclonal antibodies (Ortho Diagnostics, Cambridge, Mass) BG1, BG2, BG3, BG4, BG5, BG6, Bg7, and BG8, reactive to precursor type 1 chains; A group (type 1 and 2 chains); B group (type 2 chains), H-1; Le^a ^(type 1 chain), Le^b ^(type 1 chain), Le^x ^(type 2 chain), Le^y ^(type 2 chain) determinants, respectively. The working dilution was 1/40 for BG1, BG3, BG5 and BG6, and 1/200 for BG2, BG4, BG7 and BG8, with incubation of all the primers overnight, followed by the avidin-biotin complex method.

Similar to previous studies, we included four biotinylated lectins (Sigma Chemical Co, St Louis, Mo) from Ulex Europaeus Agglutinin I (UEA I), Sophora Japonica A (SJA), Dolichos Biflorus (DBA) and Tetragonolobus Purpureas (LTA) with the following blood group specificities: UEA I, O (H); DBA, A1>A2; SJA, B>A>O (H) and LTA, O (H) [13,14].

In addition, we used specific monoclonal antibodies NC-p53-DO7 and NCL-PCNA (Novocastra Laboratories Ltd., Newcastle upon Tuyne, UK). Titration of both antibodies without antigen retrieval was carried out. The best results were obtained at 1/100. Then an alkaline phosphatase method chromogen kit (Fast RedTM) was used. The IHC results were interpreted from each case, evaluating the percentage of reacting cells, and the intensity of the staining on a arbitrary scale from 0 (no staining) to 3 (strong staining).

The results of the immunohistochemical study were correlated with the amplification or not of the HPV-16 (part of the intron E6).

### Scoring and statistical analysis

For each case we tabulate the findings evaluating the positive or negative expression, their homogeneity or heterogeneity of the stains, the presence of isolated positive cells, the stain of the cilia of the secretor cells, the stain of the mucus, and the location of the stain in the epithelium or in the thickness of the lesion. The results of the immunohistochemical study were correlated with the blood group of the patients, which was obtained from the clinical charts.

In order to evaluate correlations between the staining results and clinicopathological variables, the chi-square test and the exact test of Fisher were applied at a significant level of 5%.

### DNA extraction and PCR

5–10 μm paraffin sections of each case were cut and placed in Eppendorf tubes of 1.5 ml. To avoid contamination, we used disposal knives and the microtome was washed with absolute ethanol between each block. We also followed recognized measures in order to prevent false positives. Sections of formalin-fixed and paraffin-embedded tumor specimens were used to extract DNA following the protocol described by Shibata et al. (1988) [32,33].

We dewased in two applications with xylene and absolute ethanol with a volume of 500 or 1000 μL, depending of the size of the tissue. Between each application, we centrifuged at 12,000 g for 5 min. The tisular sediment was resuspended in a buffer solution 1/50 of proteinase K (50 mM tris pH 8.5, 1 mM EDTA, Tween 20 at 0.5%) and the samples were incubated at 37° between 24 to 72 hours, depending on the cases. DNA was purified by phenol:cloroform followed by ethanol precipitation at -20° overnight (1/10 volume of 3 M Na-acetate pH 5.2 and 2.5 volumes of pure ethanol with subsequent centrifugation at full speed for 30 min at 4°C in a bench-top centrifuge).

125 ngm of nucleic acid was used for each 50 μl PCR reaction with Taq DNA Polymerase (Perkin-Elmer Hispania). Each PCR reaction contained primers for the intron E6 of the HPV-16 or Beta-globin. Beta-globin was used as a control gene. The primers had the following sequence: E6 HPV-16 H1+5'ATTAGTGAGTATAGACATTA3'; H2 -5'GGCTTTTGACAGTTAATACA3' and B-GLOBIN +5'GGTTGGCCAATCTACTCCCAGG3'; -5'GCTCACTCAGTGTGGCAAAG3'. These primers generated intro E6 and Beta-globin products of 109 and 536 base pairs (bp), respectively. DNA for the HPV-16 was amplified for 35 cycles on the thermal cycler Perkin-Elmer Cetus Intruments, Techne. (Table. 1)

**Table 1:** H1 and H2 primers for VPH 16 intron E6

**Subtype HPV**	**primers secuence (5'-3')**	**product PCR**
HPV 16	H1: **ATT AGT GAG TAT AGA CAT TA**	109 bp
	H2: **GGC TTT TGA CAG TTA ATA CA**	
HPV-16 E6	p1.- **ATGGAACAACATTAGAACAGCAATACAACAAACCGTTGTG**	

The 50 μl DNA amplification reactions for HPV-16 contained 2.5 μl of Buffer I (100 mM Tris-HCL pH 8.3, 500 mM KCl, 15 mM MgCl2, 0.01% (w/v) gelatin. Perkin Elmer Hispania), 0.15 mM of the dNTP mixture, 15 pmol of each primer, 2.5 units of Taq polymerase, and 1 μl of DNA.

Before adding the enzyme, we did a hot start at 95°C for 10 min., followed by 35 cycles: 45 sec. at 96°C for denaturalization, 45 sec at 55°C for anneling, and 50 sec. at 72°C for extension, with a step of final extension at 75°C for 10 min. A second PCR amplification was performed in the same conditions with 1 μl of the previous product as a template. The final product was detected in a 3% agarose gel electrophoresis stained with ethidium bromide. Each PCR reaction and the following hybridization contained negative and positive controls. The last one was a CIN-III, positive for HPV-16 by in situ hybridization and PCR amplification of the L1 region.

### DNA blot transfer and nonradiactive hybridization detection

For each sample, we added 120 μl of NaOH 0.5 M to 30 μl of the first amplification product and vortex. After 10 min. at room temperature, we added 150 μl of HN_4_-acetate 2 M and it was placed in ice. All this volume was transferred by a filtration process to a nylon membrane embedded in HN_4_-acetate 1 M (Boheringer Mannheim #1209299). After blotting we allowed for drying. The DNA was cross-linked on the blot membrane on a UV transilluminator (320 nm for approx. 40 sec.). Subsequently, we performed membrane hybridization with digoxigenin-11-dUTP-labeled DNA probe specific for the HPV-16 product amplified, followed by chemiluminiscence detection (DIG DNA Labeling and Detection Kit nonradiactive, Boehringer Mannheim). The probe for intron E6 of the HPV-16 had the following sequence: p1: 5'atggaacaacattagaacagcaatacaacaaaccgttgtg3'. (Table 1)

The cross-linked membranes were placed in a dry hybridization tube and prehybridized in a 5 ml hybridization solution (5 × SSC; 0.1% N-lauryl sarcosine, Na-salt; 0.02% SDS; blocking regeant) during 30 min. at 42°C. We replaced the hybrization solution with a new 4 ml hybridization solution preheated at 42°C and added 1 ml of 2 pmol of digoxigenin-11-dUTP labeled DNA probe previously denatured at 95°C for 5 min. The tubes were placed in a hybridization for over 16 hours at 42°C, after which we washed the membranes twice in a rotating tube holder for 5 min. at room temperature in a solution 2 × SSPE, 0.1% SDS. Then we replaced it with a 3 M TMAC solution (50 mM Tris pH 8.0, 2 mM EDTA-Na_2_, and 0.1% SDS), washing during 15 min at 61°C.

We developed the membranes by chemiluminescent detection, consisting of the following steps: 1) Wash with Buffer 1 for 5 min. 2) Wash with Buffer 2 for 30 min. 3) Incubation with the antibody conjugate anti-digoxigenin alkaline phosphatase in Buffer 2 for 30 min. 4) Two washes with Buffer 1 for 15 min. 5) Wash with Buffer 3 for 5 min and 6) Incubation for 5 min with Buffer 3 with AMPPD (0.1 mg/ml) in a dilution of 1:100 (Buffer 1: 0.1 M maleic acid; 0.15 NaCl; pH 7.5; Buffer 2: 10% Blocking regeant – Boehringer – in Buffer 1; Buffer 3: 0.1 M tris-HCl, 0.1 M NaCl, 50 mM MgCl_2_, pH 9.5).

After withdrawing the excess AMPPD, we placed the membranes in a clean hybridization bag and incubated them at 37°C for 15 min. Finally a film was exposed with the membranes at room temperature for few seconds.

We also dehybridized the membranes and attempted a second hybridization at 49°C. The results of this second hybridization were similar to the first one.

**Figure 1:**
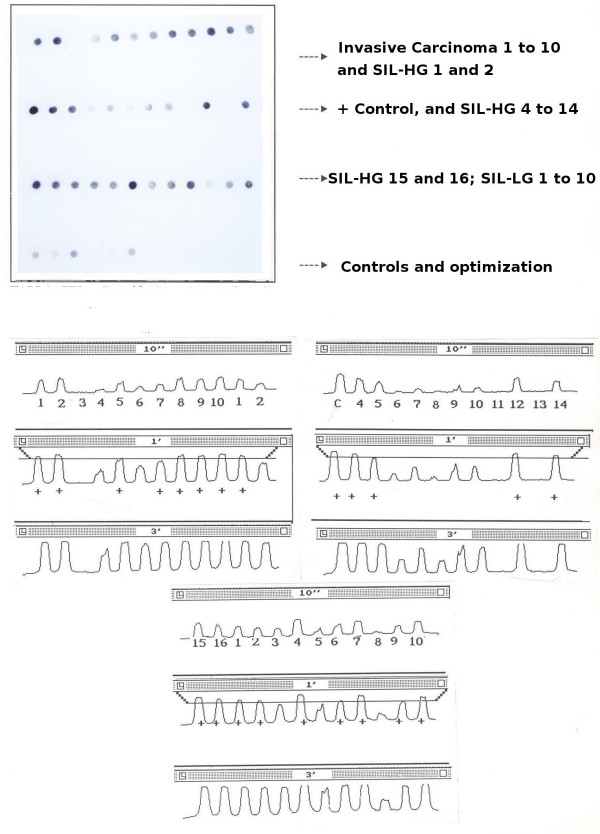
PCR – Dot-blotting. Results of the amplification from intro E6 of the HPV-16 and its specific hibridization.

The autoradiographies were scanned and studied by densitometry to generate a curve of grays for each signal. With the control cases we determined a cut-off line to establish semiquantitatively the positivity of the test (Figure 1).

## Results

**Figure 2:**
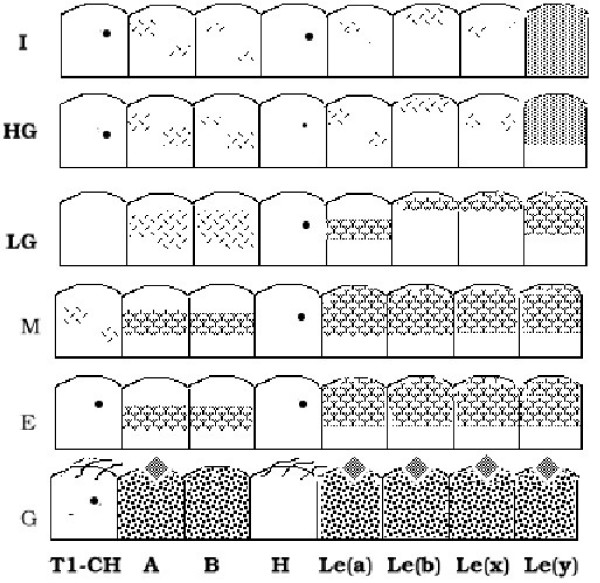
Summary of the staining profiles for anti-blood group monoclonal antibodies of the areas of cervical mature metaplasia, LG-SIL, HG-SIL and carcinomas. White areas indicate negative staining in the epithelium; dotted areas, positive staining and the localization of the staining in the epithelium. S: Simple endocervical epithelium, G: Pseudoglandular epithelium, E: Squamous exocervical epithelium, M: Mature metaplasias, LG: LG-SIL, HG:HG-SIL, I: Invasive Carcinomas. Diamon: Mucinous stain. Cilia: Cilia stain. Singel dot: ocassional positive cells.

The results are summarized in Tables 2 through 6 and in Figure 2. The eight antigens were expressed in the cytoplasmatic membrane in the case of the squamous epithelium, and in the cytoplasm/mucus of the pseudoglandular lumen, in the case of the simple epithelium. (Table 2, Table 3, Table 4, Table 5, Table 6).

**Table 2:** Summary of cases included in the study

**Blood Group**	**n**	**LG-SIL**	**HG-SIL**	**Carcinoma**
**A**	18	5	8	5
**AB**	2	1	1	0
**0**	16	4	7	5

**Total**	36	10	16	10

**Table 3:** Summary of results of the expression of antigens T1-CH, A, B and H-1.

	**T1-CH**	**A**	**B**	**H**
**Simple Epithelium**	2[27] 7.4%	11[28] 39.2%	2[29] 6.8%	2[30] 6.6%
**Epit. pseudoglandular**	16[30] 53.3%	15[29] 51.7%	2[29] 6.8%	12[31] 38.7%
**Squamous Epithelium**	2[35] 5.7%	20[35] 57.1%	10[36] 27.7%	6[35] 17.1%
**Metaplasia**	3[20] 15%	12[20] 60%	6[19] 31.5%	4[20] 20%
**LG-SIL**	0[10] 0%	4[9] 44.4%	6[9] 66.6%	1[9] 11.1%
**HG-SIL**	2[16] 12.5%	9[16] 56.25%	4[16] 25%%	4[16] 25%
**Carcinomas**	1[10] 10%	3[10] 30%	6[10] 60%	1[10] 10%

* Fist number: positive expression. Second number among braquest: Total of cases assesed

**Table 4:** Summary of results of the expression of the antigens Lewis.

	**Le**^a^	**Le**^b^	**Le**^x^	**Le**^y^
**Simple Epithelium**	17[23] 73.9%	19[27] 70.3%	18[27] 66.6%	23[26] 88.4%
**Epit. pseudoglandular**	19[27] 70.3%	20[29] 68.9%	22[29] 75.8%	27[29] 93.1%
**Squamous Epithelium**	20[33] 60.6%	27[34] 79.4%	26[35] 74.2%	29[35] 82.8%
**Metaplasia**	11[16] 68.7%	12[16] 75%	11[14] 78.5%	10[13] 76.9%
**LG-SIL**	4[10] 40%	7[10] 70%	5[10] 50%	7[10] 70%
**HG-SIL**	11[16] 68.7%	13[16] 81.25%	9[16] 56.2%	11[16] 68.7%
**Carcinomas**	**3**[10] **30%**	5[10] 50%	**2**[10] **20%**	**8**[10] **80%**

* Fist number: positive expression. Second number among braquest: Total of cases assesed

**Table 5:** Summary of results of the expression of receptors of lectins.

	**UEA I**	**SJA**	**DBA**	**LTA**
**Epit. pseudoglandular**	24[27] 88,8%	13[27] 48,1%	13[26] 50%	7[25] 28%
**Squamous Epithelium**	26[34] 76,4%	21[35] 60%	23[32] 71,8%	17[33] 51,5%
**Metaplasia**	7[11] 63,6%	8[12] 66,6%	7[14] 50%	3[14] 21,42%
**LG-SIL**	9[9] 100%	5[9] 55,5%	5[9] 55,5%	4[10] 40%
**HG-SIL**	9[15] 60%	9[15] 60%	9[15] 60%	8[15] 53,3%
**Carcinomas**	8[10] 80%	2[10] 20%	8[10] 80%	6[10] 60%

* Fist number: positive expression. Second number among braquest: Total of cases assesed

**Table 6:** Coexpression of Different Lewis Antigens and ABH Blood Group.

	**N. Lessions**	**%**
**Group I (Secretor ABH+, Lewis a, b +)**	15	41.60%
**Group II (Non secretor ABH-, Lewis a, b +)**	13	36.10%
**Group III (Secretor ABH+, Lewis a, b -)**	2	5.50%
**Group IV (Non secretor ABH-, Lewis a, b -)**	6	16.60%

The antigenic expression of the vessel endothelium was useful as an internal control. Generally, we observed conformity among such expressions and the blood group of the patients that already we knew. As in our previous studies, we found a stronger staining and a higher number of positively reacting cells were observed in both normal and neoplastic tissues, stained with monoclonal antibodies [14].

As expected, we obtained polymorphic results, given the expressive variations of the ABH blood group according to the phenotype of each individual. In addition, within each group we found individual variations in relation to the epithelial expression in accordance with their secretor status. On the other hand, the results for the expression of the Lewis antigen group were less polymorphic. In table 6 we have classified the cases according to their secretor character and the expression of the determinant antigens Le^a ^and Le^b^.

### Normal tissues

The squamous exocervical epithelium as well as the simple endocervical epithelium expressed the ABH and Le antigens in the exocervix at the level of the cytoplasmic membrane and in the endocervix in the cytoplasm and mucus of the pseudoglands.

**Figure 3:**
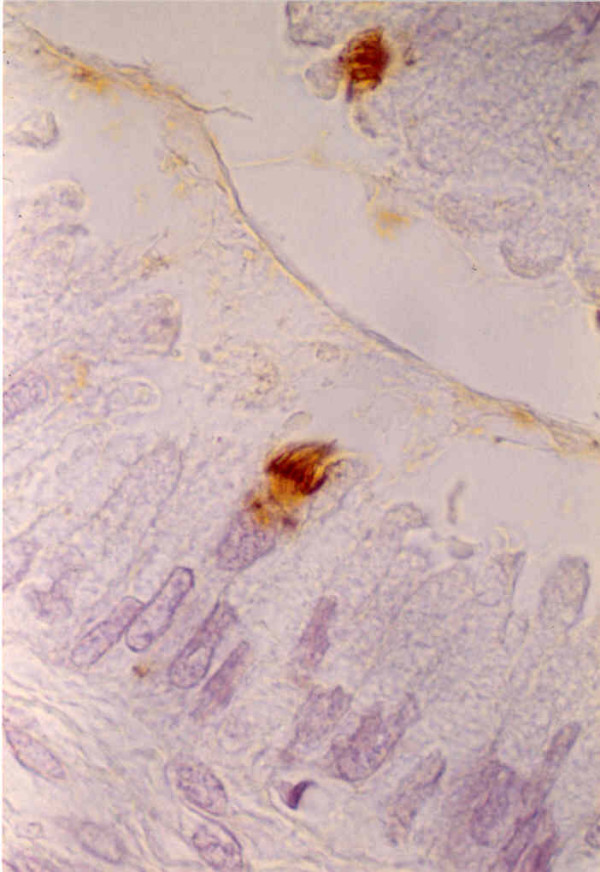
Detail of the ciliary expression of MoAb BG1 (T1-CH).

The number of positive stained cells for A and B antigens in the squamous epithelium was higher than the basal cells and mainly in the middle of the epithelium with a honeycomb pattern. Invariably, the basal cells were negative for any antibody. T1-CH and H antigens were negative in both epithelia, with only occasional expression areas in 2 of 35 cases and 6 of 35, respectively. However, in the endocervical epithelium, the ciliary processes were shown in a higher positive percentage for precursor chains as well as for the H antigen (16 of 30 and 12 of 31 respectively) (Figure 3).

Lewis antigens, when positive, were generally expressed in almost the whole thickness of the exocervical epithelium except for the basal row. The endocervix expressed Lewis antigens both in the simple epithelium and in the mucus of the pseudoglandular lights: 15 of 19 positive cases for Le^a ^were phenotype Le ^(a+b-)^(78.9%), 22 of 29 (75.8%) were Le^b^, 22 of 29 Le^x^, and 27 of 29 Le^y^.

Both the squamous and simple epithelium expressed the ABH and Lewis antigens. In the exocervix, the expression was in the membrane of the cytoplasm and, in the endocervix, in the cytoplasm and mucus of the pseudoglands.

### Areas of Mature Metaplasia

20 areas of metaplasia were available for the study in the cervix of 6 and 14 patients bearing LG-SIL and HG-SIL respectively. Three cases expressed precursor chains T1-CH and four cases the antigen H. In one of these cases we observed a heterogeneous expression of both antigens. All the patients belonging to blood group A and AB expressed A antigens with the same pattern in their normal tissues and the areas of metaplasias. The same results were seen with Lewis antigens. 11 of 16 metaplasias were positive for Le^a^ and Le^b^, 11 of 14 expressed Le^x ^and 10 of 13 Le^y^. In short, we have not found differences in the glycosylation patterns when comparing the normal exocervical epithelium with the areas of mature metaplasia.

### LG-SIL

We have not observed relevant differences in the exocervix among the patterns of expression in comparison with the LG-SIL for the ABO and Lewis blood group antigens.

Precursor chains T1-CH and antigen H were not expressed by immunohistochemistry, coinciding with the absence of expression in the squamous epithelium. We found only one of 10 cases with occasional expression fields for H1. A and B antigenic determinants, when expressed in the LG-SIL, coincided with the blood groups and the pattern of expression seen in the rest of the epithelium.

**Figure 4:**
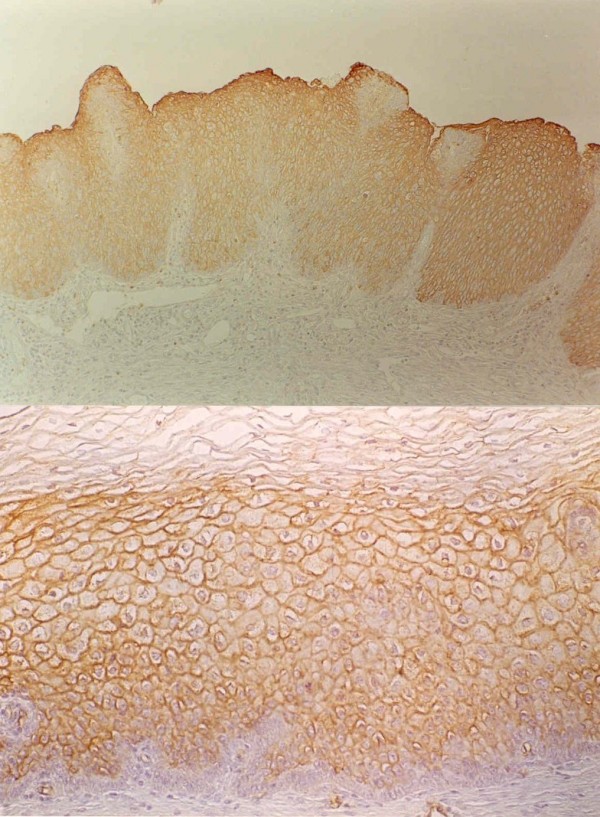
Up: Squamous intraepithelial lesion of low grade (Condyloma). Expression of MoAb BG6 (Le^b^) over the basal layer. Down: Squamous intraepithelial lesion of low degree (Flat condyloma). Uniform expression of MoAb BG2 (A) over the basal layer that is negative.

When the basal stratum was hyperplasic, this was invariably negative for the eight antigens with displacement of the antigenic expression to the most superficial layers. (Figure 4)

### HG-SIL

We have observed an increasing expression of precursor chains T1-CH and H antigen among the HG-SIL compared with the epithelia and lesions described up until now. 2 out of 16 cases (12,5%) expressed T1-CH and 5 out of 16 cases (31%) expressed H antigen. The H antigenic expression in HG-SIL was higher in the case of patients belonging to the O group (4/7 of the O group, 1/8 of the A group). In the patients in the A group with HG-SIL, we found undoubtful fields of precursor chain expression.

For the A, B, Le^a ^and Le^b ^antigenic determinants, we found significant changes of expression compared with the normal epithelium and the high-degree lesions.

**Figure 5:**
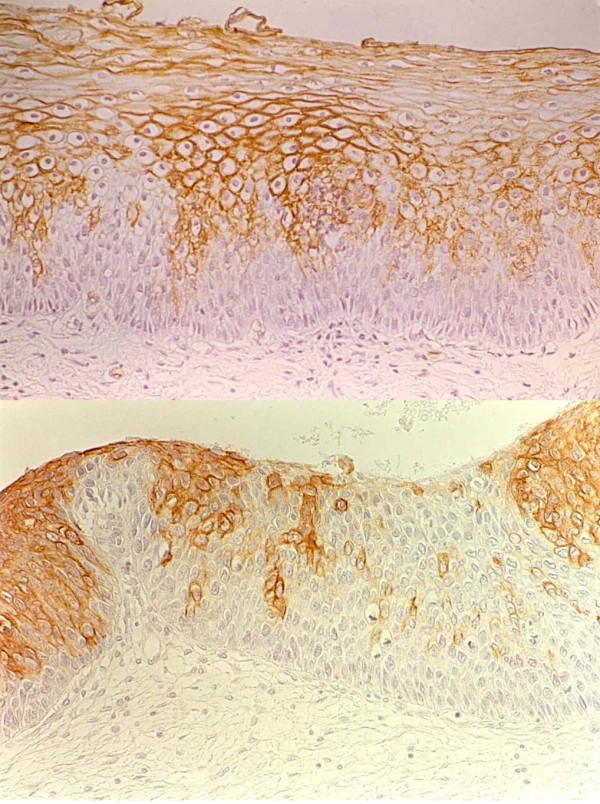
Up: CIN I with morphologic changes related to infection by HPV. Absence of expression of the MoAb BG2 (A) in the basal and parabasal layers, and expressión of this one Ab in the middle of epitelium. Down: Squamous intrapithelial lesion of the high grade. Area of heterogenous expression of MoAb BG6 (Le^b^) in a CIN III. Focal losses of expression.

**Figure 6:**
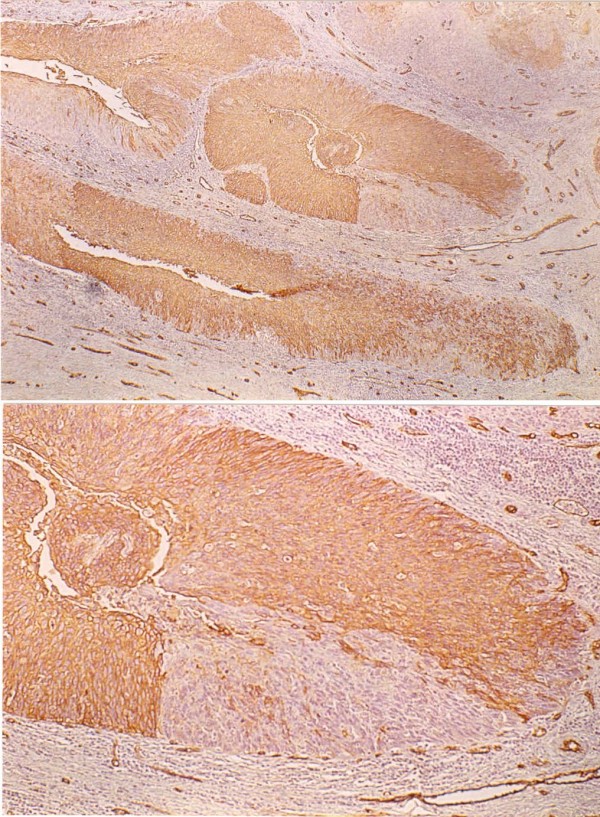
Up: Extension of CIN III in the pseudoglands that show a cellularity with heterogenous expression and diminution of the expression of MoAb BG2 (Blood Group A). Down: Detail of the same lesion where an abrupt cut of expression of MoAb BG2 is observed. In addition the expression of this Ab in the endothelial lining of small capillares can be appreciated like internal control.

All the cases with A phenotype expressed this antigenic determinant in high-degree lesions, but with a certain tendency towards a heterogeneous pattern of expression and weaker stains. 5 out of 9 cases (55,5%), positive for BG2 in normal epithelia, lost their expression in areas of HG-SIL, some with sharp losses of expression and without a gradual transition from the positive fields. Concerning the B antigenic determinant, we found four cases that expressed this antigen (4/16 25%). Three of these cases showed heterogeneous patterns. Two were patients from the O group and another patient belonged to the AB group. These four cases also expressed the same antigen in the squamous epithelium and, therefore, we cannot consider them aberrant expressions. Among these cases, we also observed focal losses of antigenic expression. (Figure 5, Figure 6)

**Figure 7:**
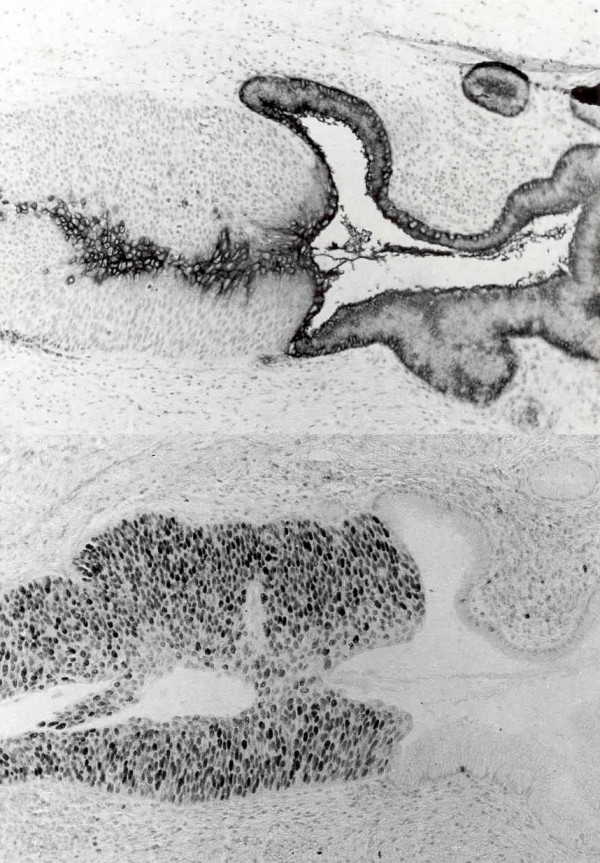
Up: Pseudoglandular endocervical with positive expression for BG5 (Le^a^) and CIN III invasion with lossess of peripheral expression of the same Ab. Down: The same cut with positive expression for PCNA in a 85% of cellularity, preferably in the periphery where the BG5 (Le^a^) was not expressed.

7 out of 11 cases -63%- positive for Le^a ^antigen had uncompleted losses of expression of this antigen (Figure 7a). Five negative cases for Le^a ^did not express this antigen in the normal epithelia 3 was Le^(a-b-) ^and 2 Le^(a-b+) ^phenotype.

### PCNA, p53 and PCR amplification of HPV16 (part of the E6 intron)

The PCNA and p53 antigens, when expressed, were seen in a nuclear pattern. In the normal exocervical epithelium, PCNA was observed to be positive in the deepest layers, corresponding with the basal and the first parabasal row. This expression was intense (++) and concerned 5% of the cellularity (index of proliferation). Only occasionally did we find some positive cells for p53.

In the endocervical epithelium, we found neither an expression for PNCA nor of p53, with the exception of some isolated cells. We have not seen differences of expression for these antigens between the mature metaplasias and normal epithelia.

7 to 10 SIL-HG cases had an increased expression of PCNA with an average proliferative index of 14,7%. This expression for PCNA was limited to the suprabasal cellular hyperplasia. On the other hand, we have not observed an expression for this antigen in those morphologic changes recognized as related with HPV infection (koilocitos, disqueratosis and atypical mitosis). p53, however was found almost positive in some scattered occasional cells.

**Figure 8:**
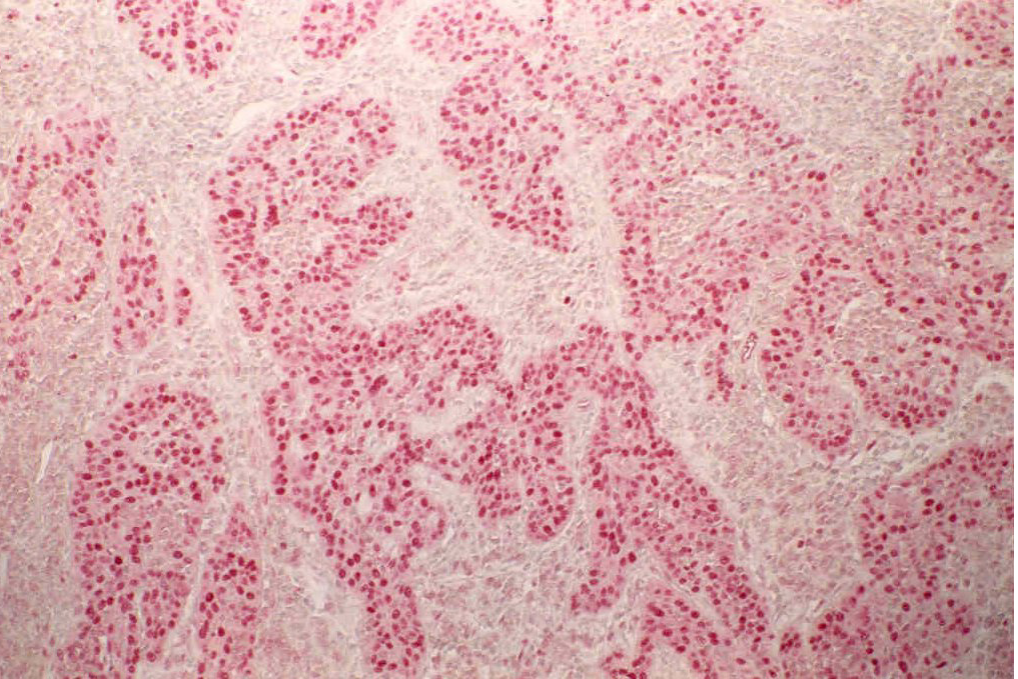
Expression of PCNA in a 100% of the cellularity of an invading squamous carcinoma.

Concerning SIL-HG lesions with invasive carcinomas, the average index of proliferation was significantly higher: 60,35% and 85% in invasive carcinomas (Figure 7b, Figure 8).

Only in one case of invasive carcinoma was p53 found over-expressed with 40% positive cell. In this case, PCNA expression presented a proliferative index of 40%, clearly inferior to the other cases.

Amplifying by PCR and with subsequent hybridization in dot-blot, we identified 7 positive cases for the gene E6 of the HPV-16 of 10 SIL-LG; 7 cases of 15 SIL-HG and 7 out of 10 invasive carcinomas (21/35 lesions).

Finally, grouping the different cases according to the positivity or negativity for HPV-16, we have found a higher proliferative PCNA index in those positive cases for the virus versus the negative cases (p < 0,05). SIL-LG: 18,28% proliferative index in HPV16(+) lesions, and 4% in HPV16(-) lesions. SIL-HG: 69,16% proliferative index in HPV16(+) lesions, and 61,42% in HPV16(-) lesions. Invasive carcinomas: 91,42% proliferative index in HPV16(+) lesions, and 62,5% in HPV(-) lesions.

## Discussion

We have compared the expression of different isoantigenic determinants of ABH and Lewis blood groups in uterine cervix tissues where we have normal epithelium and a spectrum of preneoplasias and neoplasias, from low-grade lesions (morphologic changes of epithelium related with HPV infection, condylomas and CIN I) to the invasive carcinoma.

These antigens resisted fixation with saline formaline very well. In order to compare the results with the monoclonal antibodies used, we employed different types of lectines: *Ulex Europeaus I*, *Lotus Tetragonolobus*, *Dolichus Biflorus*, and *Shopora Japonica Agglutinins *[34].

The results of both methods permitted us to observe a better stain definition when we used the MoAbs, although the patterns of stain tended to be similar in both.

This work stands in the fact that the study of cellular glycoconjugates can provide valuable information concerning the events related with neoplastic cellular transformation. Accordingly, much evidence shows that in different neoplasias the phenomena of glycosylation of certain molecules of the cellular membrane show modifications that could play an important role in relation to their malignant behavior [35].

The first works in which we began to observe those alterations date from the beginning of the 1930s [36] (Hirschfelt 1929 and Thomsen 1930). Kovarick, Davidsohn and colleagues 1968, 1969, with immunofluorescence techniques and mixed cellular reaction agglutination (MCRA), were the first authors to describe that uterine cervix neoplasias showed losses of expression of specific antigens of ABH blood groups. This fact was interpreted as a manifestation of the function of dedifferentiation analogous to morphological dedifferentiation and was related, also from the first studies, to a greater incidence of metastases. The same findings were found in adenocarcinomas of the gastrointestinal tract, ovary, tumors of transitional cells originating in the urinary tract, and in squamous carcinomas of the skin, tongue and larynx [37,17].

Likewise, since then it was recognized that in the sequence: metaplasia, dysplasia and carcinoma in situ of the cervix, a good model is reproduced in which the alterations of the isoantigen ABH expression can be followed.

Several authors have confirmed the findings of Kovarik and Davidsohn in the cervix [18,36,38]. Bonfiglio and colleagues were the first in publishing a work using, in addition to the immunofluorescence technique, an immunoperoxidase technique for the determination of the A and B isoantigens [39].

Mambo observed that 33% of the condyloma acuminatum of the uterine cervix had complete losses of some of the blood group ABH isoantigens, and partial losses in 47% [40]. We could not confirm those findings, although the number of cases in our study is smaller and our objective was not centred on low-grade lesions.

The pattern of expression of the ABH antigens in the middle of the epithelium that we have observed was previously described by Stubbe Teglbjang and colleagues using immunofluorescence. These authors found that the exocervical epithelium had a progressive pattern in the expression of the blood group isoantigens, with expressions in the basal and parabasal cells of N-Acetyllactosamine, of the antigen H in the parabasal and in the cells of the low spinosus stratum, and of the A antigen in cells of the high spinous stratum. They also observed that the premalignant and malignant lesions had irregular decreases in the content of the antigens A and H, with an almost complete loss of them, and precursor chain accumulation [41].

Following a technique using immunohistochemistry with the avidin-biotin complex, we have not been able to observe a clear accumulation of precursor chains although we have found significant results in the loss of expression of the A antigen.

To and colleagues have described that the expression of the blood group antigens, in combination with the depth of the tumoral invasion, could serve as a predictor of survival in cervical carcinoma [42]. In the same direction, Sakamoto and colleagues observed a greater frequency of complete loss of the antigen H in small cell carcinomas, which have also a worse survival to the two years, versus to the squamous carcinomas with a better survival [43].

The sum of this evidence has led to us to study, after our experience with the respiratory mucosa [13,14], the alterations of the glycosylation in different cervical lesions. Our findings are very similar to those from our previous studies, leading us to believe that these alterations could be a phenomenon common in different tumors with different etiological implications.

Our work, unlike its precedents, is complemented with the use of lectins, a tool that has served to control the results by means of immunohistochemistry to discard possible artefacts motivated by the fixation or the different processing of the tissues.

On the other hand, HPV infection has been strongly related to cancers of the lower female genital tract, penis, larynx, lungs and anus. It is known that the subtypes 16, 18, 45 and 56 in cervical lesions are related to a greater relative risk of developing invasive carcinomas [44,45].

Some papers point out that the percentage of PCNA positive proliferation cells is significantly higher in premalignant and malignant lesions of the uterine cervix than in nonneoplastic lesions [46].

To investigate the possible differential expression of PCNA in negative and positive lessions of the cervix for E6 HPV-16 lesions, we have studied, using immunohistochemistry and PCR, different cases from flat condylomas to invasive caricinomas. We also inquired into the overexpression of p53 in order to explain the cases with a possible alteration of this tumor supressor gen.

We did not find a significant expression of p53 as other authors have reported [47]. Only occasional basal or parabasal nucleus could be seen as positive with the DO-7 MoAB clone. However PCNA was expressed more progressively from a lower grade to the higher degrade and invasive carcinomas. Our results are consistent with the observations that the cell proliferating index as detected immunohistochemically using PCNA may be a useful parameter to indicate the degree of CIN. In addition, we found in our study a higher PCNA proliferation index in CIN III and invasive carcinomas of cervix positive for HPV-16 versus the cases that are negative for this type of virus.

## Conclusion

To sum up, the results and conclusions of this paper are as follows: (1) In the invasive cervical carcinomas, we observed a loss of expression of the Le^x ^antigen (p < 0.01). (2) Almost significant differences were observed when comparing the expression of Le^a^, and blood group A antigens in squamous epithelium and invasive carcinomas (p < 0.1). (3) With regard to the progression of the different lesions studied, we found alterations in the patterns of expression of the antigens of the ABH and Lewis blood groups. There was a tendency towards loss of expression and heterogeneous patterns in the more advanced lesions, and also overexpression of the Ley antigens. (4) With PCNA, we established a proliferative rate which tended to be greater in relation to the progression of the cervix neoplasms. (5) We found only one positive case of MoAb DO-7 protein p53 in the invasive carcinomas that produced negative results for the HPV-16 virus. (6) Using PCR techniques, we had a 60% detection rate of part of the intron E6 of the HPV-16 in all the lesions included in the study.

## Competing interests

The authors declare that they have no competing interests.

## Authors' contributions

EMR Preparation of cases, immunohistochemical investigation, molecular genetic studies, analysis of data, statistical analysis. EAF conceived of the study, participe in its design coordination and interpretation of data. All authors have read and approved the present manuscript.
